# A Lady Quelled Them

**Published:** 1884-08

**Authors:** 


### A LADY QUELLED THEM.

THE disorderly element among college-boys is not—we will suppose in charity to them—necessarily a proof that they are worse than other boys; but rather a relic of savagery in human nature, which circumstances and numbers provoke into activity. When the fun boils over too violently, students can be made ashamed of it, and the shame redeems them. Such a result was handsomely achieved, not long since, by a well-known and gifted lady when a band of thoughtless young "medics" ventured in offensive joking:

At a medical clinic at Blockley almshouse last week, says the Philadelphia *Record*, three female students occupied seats in the lecture-room, and while waiting for the lecturer, who was belated, the young men of the class indulged in some noisy demonstration, which was finally directed in the way of playful banter to the women present.

Suddenly Miss A. M. Field, one of the female students, arose, and, as she began to speak, the noise was changed to respectful silence.

"Gentlemen," she said, "I have been for eighteen years a missionary in China. The Chinese have no medical science, and superstitious rites are chiefly relied on in the treatment of diseases. All the people are in need of medical aid, but the women are the neediest.

"A Chinese woman would under no circumstances go to a male physician for the treatment of any disease peculiar to her sex. She would be prevented by her own womanly delicacy and by all the notions of modesty held by those around her.

"She would suffer life-long agony rather than violate her sense of propriety. Her father, her brothers and her husband would even let her die rather than allow her to be treated by a male physician.

"Full of sorrow for the sufferings of these women, I have been looking in Christian America to see what hope of help for them might be here. I have been glad to find that in some of our great medical schools earnest and self-sacrificing women are fitting themselves for a work of mercy in Asia and other lands.

"Unless such women learn to do such work well, there is no physical salvation for those afflicted ones. And in behalf of those women, who have no medical care while they so sorely need it, I ask from you the courtesy of gentlemen towards ladies who are studying medicine in Philadelphia."

As Miss Field sat down she was greeted with a cheer, and a member of the class arising assured the ladies in a very gallant speech that no annoyance to them was intended. The timely remarks of Miss Field had touched the inborn courtesy of the young men, and taught them a lesson they will probably never forget.

